# Integrating Rehabilomics into the Multi-Omics Approach in the Management of Multiple Sclerosis: The Way for Precision Medicine?

**DOI:** 10.3390/genes14010063

**Published:** 2022-12-24

**Authors:** Bruno Bonnechère

**Affiliations:** 1 REVAL Rehabilitation Research Center, Faculty of Rehabilitation Sciences, Hasselt University, 3590 Diepenbeek, Belgium; bruno.bonnechere@uhasselt.be; 2 Technology-Supported and Data-Driven Rehabilitation, Data Science Institute, Hasselt University, 3590 Diepenbeek, Belgium

**Keywords:** rehabilitation, genes, gut microbiome, precision medicine, rehabilomics, technology-supported rehabilitation

## Abstract

Over recent years, significant improvements have been made in the understanding of (epi)genetics and neuropathophysiological mechanisms driving the different forms of multiple sclerosis (MS). For example, the role and importance of the bidirectional communications between the brain and the gut—also referred to as the gut-brain axis—in the pathogenesis of MS is receiving increasing interest in recent years and is probably one of the most promising areas of research for the management of people with MS. However, despite these important advances, it must be noted that these data are not—yet—used in rehabilitation. Neurorehabilitation is a cornerstone of MS patient management, and there are many techniques available to clinicians and patients, including technology-supported rehabilitation. In this paper, we will discuss how new findings on the gut microbiome could help us to better understand how rehabilitation can improve motor and cognitive functions. We will also see how the data gathered during the rehabilitation can help to get a better diagnosis of the patients. Finally, we will discuss how these new techniques can better guide rehabilitation to lead to precision rehabilitation and ultimately increase the quality of patient care.

## 1. Introduction

Multiple Sclerosis (MS) is one of the most prevalent neurological disorders, affecting over 2.5 million people worldwide. It is an autoimmune and demyelinating central nervous system (CNS) condition [1]. MS is associated with a variety of symptoms, such as physical performance impairment and tiredness [2], anxiety and depression [3], and cognitive impairments [4]. To date, the pathophysiology of MS is still largely unknown, although it is widely assumed that immune responses to multiple myelin antigens cause disease development [5].

One of the most probable mechanisms for MS pathology has been proposed to be cytokine and adipokine imbalance in the CNS and peripheral blood circulation in persons with MS [6]. Interestingly, it has been shown that physical activity and rehabilitation exercises may be associated with increasing IL-10 levels and decreasing TNF-α levels in people with MS (pwMS) [7].

Major advances have been made in the prognosis (thanks to the multi-omics approaches) and in the management of pwMS, largely due to the development of technology-supported rehabilitation [8]. Omics technologies are high-throughput biochemical assays that test molecules of the same kind in a biological sample extensively and concurrently [9]. The goal of multi-omics, also known as integrated omics, pan-omics, and trans-omics, is to merge two or more omics data sets to help in data processing, visualization, and interpretation to discover or further understand the mechanism of biological activity [10].

Rehabilitation, which involves counseling and symptomatic therapy, is now thought to be one of the most effective treatments for maintaining optimal functioning and, therefore, quality of life in people with MS (pwMS) [11].

The disease’s intricacy, the difficulties in establishing the best treatment, and the wide variety of symptoms necessitate a holistic approach to the patient that includes both pharmacology and neurorehabilitation [11]. The availability of multi-omics data has transformed medicine and biology by opening up options for integrated system-level methods [12].

However, there is currently a lack of integration of the data and information from the medical world (e.g., multi-omics) and rehabilitation specialists, although this information could be useful in selecting the most appropriate rehabilitation techniques. In looking at the problem from the opposite direction, there is also a lack of communication and a lack of use of the data (e.g., ranges of motion, smoothness, strength) obtained during the rehabilitation towards the medical world to obtain a more precise diagnosis and follow-up of patients since have to undergo many rehabilitation sessions. It is, therefore, possible to perform longitudinal follow-up.

In the paper, we will first discuss the latest finding of genetics analysis as well as the modification of gut microbiota observed in pwMS. Then we will discuss the different rehabilitation strategies and then see how the information from the gut microbiota can be used as an indicator of the progress of the patients during the rehabilitation process. Finally, we will discuss how the information collected during the rehabilitation process can be used to integrate more functional information in the follow-up and management of pwMS.

## 2. The Multi-Omics Approach

**Figure 1:**
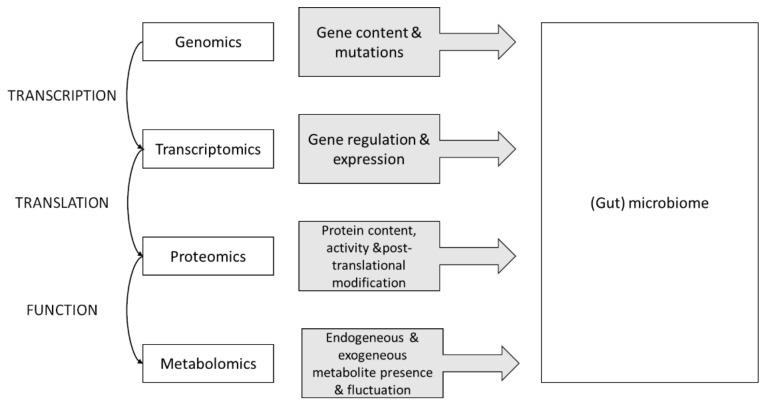
Schematic representation showing the integration of the different omics technology (white boxes on the left) and their roles and interactions (grey boxes, center) with the gut microbiome, adapted from [13,14,15].

Multi-omics is a novel approach in which data sets from various omics groups are combined during analysis. The various omics strategies analyzed include individual components: genomics, epigenomics, transcriptomics, proteomics, metabolomics and phenomics [13], as well as the complex relationship between these components (see Figure 1) [14]. A multi-omics method allows us to answer fundamental biological issues such as which genes are expressed into RNA (i.e., transcriptomics) and translated into proteins (i.e., boproteomics), as well as which metabolites are present or vary under different situations (i.e., metabolomics). The rapid development and democratization of the technologies (e.g., DNA and RNA (next-generation) sequencing [15], single-cell sequencing [16], DNA methylation analysis [17], Quantitative Mass Spectrometry-Based Proteomics [18] and metabolomics [19]) coupled with the development of bioinformatics allows the rapid development of this field. Major advances have been made thanks to the integration of this information.

First, we will provide a brief summary of the current findings in genetics.

A total of 233 statistically independent and genome-wide significant associations with MS susceptibility have been discovered in the most recent GWAS study [20]. The team discovered that the major histocompatibility complex (MHC) comprises 32 connections, one of which is the first MS locus on a sex chromosome, which is located on chromosome X. The autosomal non-MHC genome comprises the remaining 200 associations. A large number of these loci are probably definitely real susceptibility loci. The genome-wide and suggestive effects account for 48% of the estimated MS heritability [20]. However, recently, environmental variables, rather than purely genetics, have been proposed as important predictors of vulnerability [21]. Therefore, we will mostly focus on the microbiome, one of the most promising developments in this field.

The role and importance of bidirectional communications between the brain and the gut—also known as thegut-brain axis—in the pathogenesis of various central nervous disorders has grown in recent years and is likely one of the most promising areas of research [22]. The gut microbiome is made up of many different microorganisms: approximately 1000 bacterial species and 7000 bacterial strains have been identified, totaling 10^13–^10^14^ different microorganisms in the gut [23]. The gut–brain axis is a bidirectional communication axis that includes, among other things, the intestinal microbiome, the intestinal barrier, intestinal inflammation, and the intestinal/systemic/brain immune systems [24]. The gut–brain axis is involved in both normal and pathological central nervous system function [25,26], and gut microbiota alterations are associated with modulation in the immune system markers [27].

A recent review summarized the quantified evidence of gut microbiome in several neurological disorders, including MS [28]. Ten studies involving a total of 307 pwMS and 311 healthy controls were included. Despite the small number of patients and controls, there was a relatively high consistency seen at the genus level involving 11 associations in the different studies (*Actinomyces, Akkermansia, Bifidobacterium, Coprococcus, Dialister, Dorea, Faecalibacterium, Haemophilus, Megasphaera, Paraprevotella,* and *Slackia*) and six associations in the opposite direction—the direction of the modification was found inconsistent between the different studies (*Butyricicoccus, Clostridium, Gemmiger, Parabacteroides, Phascolarctobacterium,* and *Prevotella*) [28]. From a neurophysiopathological point of view, *Akkermansia* is certainly one of the most important microbiota. *Akkermansia* is found in the mucus layer of the large intestine [29], where it is involved in the process of maintaining the integrity of the intestine and degrading mucin [30]. *Akkermansia* can stimulate dendritic cells to produce TGF-β and interleukin 6 (IL6) and 1 (IL1), activating regulatory T Cells (Tregs), which may be relevant for the pathogenesis of MS. Changes in gut microbiota are connected to CNS inflammation in neurological disorders and MS is a neuroinflammatory condition.

Interestingly important changes in gut microbiota composition have also been highlighted in depression [31], a common symptom of MS (with an increased incidence of up to 71% compared to the control population) [32]. It is of note that there is no overlap in the gut microbiome notification in MS and depression [28,31], nor at the genus level [33]. In this recent bidirectional Mendelian Randomization study, the authors did not find a significant risk for the development of MS in persons carrying variants associated with depression or for risk of depression in individuals who are genetically susceptible to MS [33].

Concerning fatigue, another frequent symptom of MS, the evidence is less clear in MS patients as most of the studies are currently performed in cancer patients [34].

## 3. (Conventional) Rehabilitation

The bacteria that live in the gut tend to be sensitive to environmental influences and interventions such as changes in food and medicine, as well as physical exercise [35,36]. Engaging in regular physical activity is a helpful intervention for pwMS. This intervention provides a number of benefits, including improved brain function and the status of the immune system, as well as an increased ability to carry out day-to-day activities [37,38]. Positive modifications of the gut microbiota have also been linked in a number of studies to the frequent participation in physical exercise in both human subjects and animals [39].

Rehabilitation strategies are a group of therapies aimed at improving functioning and reducing impairment in persons with chronic diseases who interact with their environment. Person-centered rehabilitation indicates that the therapies and techniques chosen for each individual should be based on their goals and preferences [40]. Rehabilitation can be provided in a variety of settings, including inpatient or outpatient hospital settings, private clinics, and community settings such as a person’s home. Rehabilitation professionals include physiotherapists, occupational therapists, speech and language therapists and audiologists, orthotists and prosthetists, clinical psychologists, physical medicine and rehabilitation physicians, and rehabilitation nurses, among others [41]. Rehabilitation and exercises have been found effective in improving MS effects such as fatigue [2], activity and participation [42], and therefore the quality of life [43] of pwMS.

Clinical outcomes are often determined by observing participants’ motor actions (e.g., to capture motor impairments and functional limitations using specific tests and scales such as the Expanded Disability Status Scale [EDSS] [44]). Regrettably, these evaluations are time-consuming and impracticable to do on a consistent basis during the intervention period. Too often, outcome measurements are gathered only at baseline and discharge. This is a concern since the absence of longitudinal data precludes rehabilitation professionals from exploring the possibility of changing the intervention in order to maximize motor progress. The development of new technologies is one solution and could eventually lead to the development of a hybrid formula between sessions performed with clinicians and technology-led sessions.

## 4. Technology-Supported Rehabilitation

**Figure 2:**
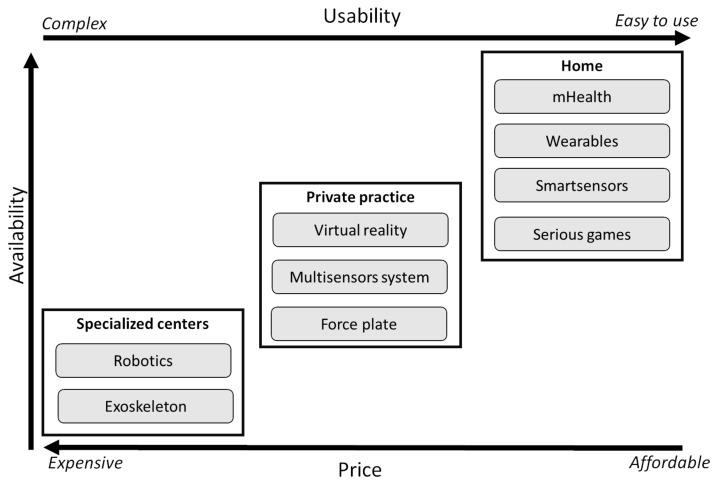
Schematic representation of technology-supported rehabilitation. Note that several technologies can be used in different categories (namely specialized centers, private practice or at-home). The x-axis represents the price and ease of use of the technology. For example, robotics technology is expensive (>$100,000) and requires trained staff, while mHealth apps prices are usually between $1 and $50 per year and can be used by the patients themselves without help or support. On the y-axis is the availability of the devices for both patients and clinicians.

The rapid development of technology and computer science has changed our environment and our way of life enormously over the last decades. The use and implementation of new technologies to assist and improve physiotherapy and the rehabilitation process is called technology-supported rehabilitation. The technology that is, or can be used, in rehabilitation can be divided into three categories: (i) high-tech devices whose price and complexity of use limit their use in specialized centers (i.e., robotic gait rehabilitation [e.g., Lokomat^®^ from Hocoma, Lexo^®^ from Tyromotion], exoskeleton [EksoNR^®^ from eksoBIONICS]), (ii) devices that can be used by the clinicians in their daily practice (i.e., serious games exercises with or without virtual reality headset [e.g., Jintronix, CorpusVR]), and iii) systems and solutions that can be used by the patients alone at home (i.e., mobile health applications [e.g., Physitrack, MoveUP, Physiotools]) (Figure 2).

Rehabilitation can be facilitated by existing technology and help both the patients and the clinicians. Another great potential of using technology to support rehabilitation is that different measurements can be taken while patients are performing the rehabilitation, either in the clinic or at home. These set of measurements can be used as continuous outcomes (e.g., reaction time, smoothness of the motion, number of steps) to monitor the evolution of the patients during the rehabilitation process (e.g., high-intensity training, stretching, balance training) and adapt the plan according to the real needs (e.g., improving quality of life, decreasing level of fatigue) and specificities (e.g., different forms of MS) of the patients. The current development of inexpensive and portable systems using wearable sensors is gaining popularity in the rehabilitation area, and such systems can be used in daily clinics with patients to measure, for example, upper limb motion [45], hand function [46], balance [47], and gait [48].

The majority of devices allow the continuous recording of the motions performed by patients during rehabilitation exercises [49,50]. These measures are technically referred to as biomarkers. The word refers to a large subset of medical indicators ‘indicators of the normal biological processes, pathogenic processes, or responses to an exposure or intervention, including therapeutic interventions accurately and reproducibly measured from outside the patient’ [51]. Rehabilomics has been defined by Wagner as “a novel framework from which to discuss biomarkers in research and clinical care that addresses research gaps and clinical treatment needs specific to physical medicine and rehabilitation” [52]. Rehabilomics combines the systematic data collection of rehabilitation-relevant phenotypes and transdisciplinary analysis of biomarkers to better understand the biology, function, prognosis, complications, treatments, adaptation, and recovery of people with disabilities.

This physiologically based conceptual framework essentially adds an “-omic” layer to the scientific study of rehabilitation processes and results, personalizing a biomolecular approach to rehabilitation therapy aimed at improving individual recovery [53]. These analyses could be used later to adjust the dose, type and intensity of the different rehabilitation exercises at an earlier stage of the research or to decide to discontinue an intervention if it turns out that the patient cannot benefit [54].

Mobile health technologies (i.e., wearable, portable, body-fixed sensors, or home-integrated devices) that evaluate mobility in an unsupervised way on a daily basis during activities of daily living are gaining popularity as supplemental clinical assessments [55]. Due to the fact that data gathered in these ecologically valid, patient-relevant contexts capture varied and uncommon occurrences, they may circumvent the limitations of conventional clinical examinations (i.e., ceiling effect, time-consuming, cross-sectional evaluation) [56]. Remote health monitoring, based on non-invasive and wearable sensors and communication and information technology, enables patients to remain safer at home [57,58]. In addition to the safety effect, the remote monitoring of patients during activities of daily life between sessions is a significant part of the application of new technology in rehabilitation. Continuous data gathering can be used to detect considerably smaller changes in patient status (i.e., improvement or deterioration) [59] and should be utilized in research to produce more accurate and sensitive outcomes such as digital biomarkers [60]. Using digital biomarkers may monitor and gather biological, physiological, and anatomical data objectively and more continuously [61]. Moreover, persistent, objective monitoring can uncover illness characteristics not found in the clinic using classical tests [62]. Using smartphone-based digital biomarkers both for assessment and to drive the rehabilitation process is a significant area of development due to their widespread availability [63]. There are two distinct types of digital biomarkers: active (supervised) ones in which participants must complete specific tasks, such as cognitive tasks (e.g., Lumosity, BrainHQ, Peak Brain Training) [64,65], and passive (unsupervised) ones in which the outcomes are derived from the natural use of the smartphone, a technique known as digital phenotyping (e.g., Neurocast) [66]. Digital phenotyping can be used to track age-dependent cognitive and behavioral processes in MS patients and has shown good responsiveness to changes in disease activity, fatigue, and clinical disability [67].

We have seen that the technology can be used not only to increase the quality of the rehabilitation but also provides a unique opportunity to collect continuous data in a more ecological way (i.e., rehabilomics and digital biomarkers). This can be particularly relevant for rehabilitation as one of the biggest current limitations of the translation in rehabilitation is that the outcomes used in the research study, mainly subjective evaluated by the clinicians, may not be sensitive enough to detect subtle modifications of the patients because of low resolution and ceiling effect [68,69].

In the next part of the paper, we will see how we can integrate and use these data from the rehabilomics within the well-established multi-omics approach.

## 5. Toward a (More) Integrated Approach: The Precision Medicine

There is, currently, to the author’s best knowledge, only a very limited number of studies trying to integrate omics information into the rehabilitation process.

Two studies about the use of gut microbiota as an outcome of rehabilitation intervention have been recently published in the field of MS.

In a first pilot study, authors highlighted the positive effect of a high-impact multidimensional rehabilitation program of gut microbiota on a small sample of 14 pwMS. This pilot investigation reveals the possibility of a short, high-impact multimodal rehabilitation program, including neuromotor rehabilitation, sailing course, mindfulness program and Mediterranean diet, to modify MS-typical dysbiosis by lowering pathobionts levels while restocking SCFA-producing beneficial bacteria, restoring a partial eubiotics profile. As shown by lower circulating levels of lipopolysaccharide and a drop in pro-inflammatory lymphocyte populations, such alterations might offset the normal inflammatory tone seen in MS [70].

In the second study 42 pwMS were included and participated in a six month home-based rehabilitation program [71]. After the intervention, the authors showed that exercises significantly increased *Prevotella* counts and decreased *Akkermansia* counts but had no effect on circulating cytokine levels. However, *Akkermansia muciniphila, prevotella* and *Bacteroides* count changes in response to the intervention were correlated with changes in IL10 (r = −0.52, r = 0.67, and r = −0.55, respectively) [71].

These two studies show promising results and indicate that partial return of a eubiotic profile may assist in counterbalancing the inflammatory tone seen in MS, as seen by lower circulating lipopolysaccharide levels and pro-inflammatory cell populations.

**Figure 3:**
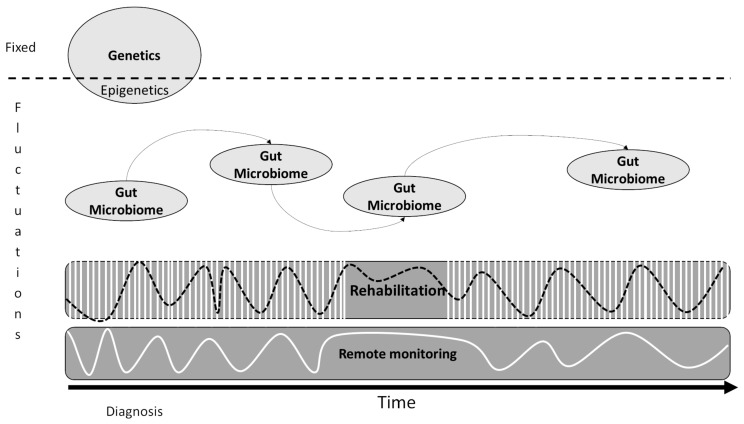
Schematic representation of the potential integration of rehabilomics data in the assessment and longitudinal follow-up of the patients over the course of the disease. Compared to other clinical measurements (e.g., genetics analysis performed once, brain imaging performed at intervals of several years, analysis of the microbiome should be performed more regularly to catch seasonal fluctuations [78]), functional data collected during the rehabilitation process and/or in the living environment can capture with more accuracy the fluctuations (e.g., changes in motor and/or cognitive functions [82]) of the patients during the rehabilitation sessions (indicated by the black sinusoidal line on the schema, vertical white lines indicated the rehabilitation sessions) and during activities of daily living (indicated by the white sinusoidal line on remote monitoring box), which is of particular importance for pwMS, since they can be done on a regular basis (i.e., rehabilitation) or continuous basis (i.e., remote monitoring).

It has been previously highlighted that multidisciplinary measurements, including clinical, functional and patient-reported outcome measures in combination with extensive patient profiling, can enhance personalized treatment and rehabilitation strategies [72,73]. Therefore, let us now discuss why the data collected during the rehabilitation process should be included in the multi-omics analysis. A schematic representation is presented in Figure 3. At the time of diagnosis, genetic information will be of interest to define the type of MS [74], and this information will not change over time (apart from epigenetics and the activation resulting in transcription). Then, most of the outcomes of interest will be subject to significant variation and fluctuation during the course of the disease. The gut microbiome is, for example, subject to variations depending on diet [75,76], constipation [77], seasons and environment [78], global and local inflammation levels [79], stress [80], and medication [81].

Concerning rehabilitation, both motor and cognitive functions will show very important variations over time (important variations during the day, week or seasonal fluctuations) [82]. Of course, if we only perform cross-sectional evaluations, the chance of missing these variations is high and therefore, the risk of over- or under-estimating the impact of the disease on the patients’ functions is high [82].

In this particular context, the rehabilitation process, and more specifically the use of technology-supported rehabilitation, provided a unique opportunity to switch the way we are gathering functional data of pwMS from a cross-sectional to a longitudinal way. Technology-supported rehabilitation has therefore gained popularity due to its ability to provide an objective and, if necessary, blind assessment, which can be automated (time saver) and allows for a measurable evaluation of motor function by taking into account the patient’s characteristics (e.g., kinematics, activity level, intensity, muscle activity, co-contraction, posture, smoothness, heart rate, stress, etc. [83]) and therapy adherence [84]. Technology allows this data collection without adding extra workload on the clinicians or patients since the data are collected automatically during the rehabilitation exercises or during activities of daily living [85], using both quantitative or more qualitative (i.e., patients’ reported outcomes) measurements [86]. Therefore, rehabilitation biomarkers are growing progressively from simple clinical behavioral measures based on quantitative scales to brain imaging and neurophysiological studies [87].

## 6. Current Challenges and Call for Actions

If the potential added values of such kinds of continuous evaluation are obvious, such as adapting treatments (i.e., pharmaceutical, rehabilitation plans) to the actual need and specificity of the patients (i.e., personalized medicine) to maximize the benefits of the intervention, building such a platform is highly challenging.

The challenges can be seen at two distinct levels: technological and human.

Let us first focus on the current obstacles and limitations related to the technology and its implementation.

First, an important investment must be carried out to acquire the rehabilitation equipment and the sensors (remote monitoring) in order to acquire the data. The price of these systems (Figure 2) varies enormously, ranging from several hundreds of thousands of EUR for the exoskeleton and robotics to a couple of dollars for mHealth applications. Note that, of course, these systems are also used for the care of the patients [8], not only for collecting data. Therefore, these investments also have a direct positive impact on the patients.

This takes us to the second most significant existing limitation: the lack of reimbursement from the social security system. The organization and involvement of healthcare systems in the rehabilitation process varies by nation, and we will not go into detail about reimbursement here. However, it is well known that financial concerns and a lack of expertise and familiarity with the use of (new) technology are two of the most significant barriers to the deployment of technology for patients, regardless of their diseases [88,89]. Another important point that could help the implementation of such technologies is the recognition of these systems as medical devices. In 2020 the FDA approved the first game-based therapeutic device for children with attention deficit hyperactivity disorders. The device is designed for usage as part of a treatment program that may include clinician-directed therapy, medication, and/or educational activities to address the disorder’s symptoms [90]. Since then, the game has been increasingly used in the United States.

Interestingly, it is of note that the COVID-19 epidemic has not only disturbed healthcare systems but has also accelerated the development, deployment, and acknowledgment of new technology, in particular, mHealth, in rehabilitation [91]. It is crucial to highlight, however, that most of the steps implemented during the crisis may be transitory, and it is anticipated that efforts in this direction will continue once the crisis has passed.

Third, with respect to data, it is important to ensure the quality of these. A distinction must be made here between data acquired in a supervised manner (with the physiotherapist during the rehabilitation) and data acquired in an unsupervised manner (remote monitoring). For the first one, since they are acquired under the control of a clinician, the quality can be checked directly [84]. For home assessment, two types of activities can be monitored: rehabilitation exercises and activities of daily living. On the one hand, for the rehabilitation exercises, a recent study highlighted the fact that when recording at home and comparing to their performance in the lab, individuals performed all activities equally. However, as compared to the physiotherapist’s demonstrations, participants completed all exercises faster, showing the necessity for a wearable device with user input that will set the tempo of the activity [92]. On the other hand, for the remote assessment (i.e., assessment during activities of daily living), since direct control of the clinicians is not possible and the activities are not as well standardized as the rehabilitation exercises [58], the assessment of the data quality is a much bigger challenge [93]. Different data sources (e.g., GPS, weather information, self-reported outcomes, quality of sleep, etc.) must be used to assess the quality of the data and evaluate the context in which these data were collected [94,95].

The next challenge is then the merging and synchronization of different data sources (i.e., the different rehabilitation technologies presented in Figure 2 but also the other omics technologies), data format and time series into one single database. Then, once the data are stored and well synchronized, important data cleaning and selection process must be carried out to extract the most relevant features from the big database [96]. Another potential limitation is the difficulty of accessing hospital medical data since these are often protected with limited outside access, and the data may be stored in different databases [97], but important efforts are being made to ease the access and use of the medical health records in pwMS [98].

However, we do not have to reinvent the wheel. Tremendous efforts have already been made in the omics world [99], so all the techniques previously developed can be easily implemented within the rehabilomics framework even with relatively small sample size studies [100].

The second group of limitations concerns the acceptance of the technology by both the patients and the clinicians.

Because most pwMS are young and are familiar with smartphones, apps, and mobile technology, familiarity with technology should not be a concern for the majority of them [101], but it can be a serious barrier for other illnesses or patient groups (e.g., older pwMS with dementia) [102]. Efforts must also be made to educate healthcare personnel since they must be fully informed of the technology and its limits in order to persuade patients to utilize it. In addition to the quality of the data (see the previous section), another important potential limitation is the low—or lack of—patient adherence to home exercises. It is indeed well known that adherence to home exercises is relatively low in physical rehabilitation (only about 30% of the patients do the right amount of exercises and repetitions) [103]. This low adherence could potentially lead to an insufficient amount of data (i.e., missing data) to successfully capture small changes in patients’ functions [104]. Fortunately, one of the most important potential advantages of the new technologies is that most of the exercises can be combined with serious games, and gamification has been shown to increase patient motivation and adherence [105].

In order to ease the implementation and use of new technology in the healthcare sector in general, and more specifically in rehabilitation, the next step would be to incorporate technological solutions into the healthcare system, including compensation for patients, education of healthcare professionals on these solutions, and integration of data obtained with the technologies in patients’ medical records. This might hasten and simplify the incorporation of these technologies into the everyday care and rehabilitation of pwMS. If successfully implemented, such kinds of highly multidimensional and longitudinal databases could be used to unravel the complex relationship between genetics, inflammations, imaging and functional data and allow us to further understand the implications of the genes in the functional pathophysiology of pwMS.

## 7. Conclusions

The multi-omics approach has drastically helped the understanding of the complex pathophysiological underlying neurodegenerative disorders such as MS. While these disciplines are still currently mainly used to develop new drugs therapy, this information should also be used to advance rehabilitation.

On the other hand, it is essential to incorporate more functional data both in basic research and in the clinical follow-up of patients. To do this, physiotherapists are on the front line, and thanks to the use of new technologies, a lot of data can be collected automatically during revalidation sessions but also during activities of daily life. It is, therefore, of the utmost importance to develop highly multi and interdisciplinary collaborations between researchers, clinicians, and data specialists to integrate these data into one single pipeline to move forward in this field.

## Data Availability

Not applicable.
